# Macular and peripapillary Choroidal Vascularity Index in children with different refractive status

**DOI:** 10.1038/s41433-023-02743-1

**Published:** 2023-09-28

**Authors:** Ziyi Qi, Xiaoxiao Liu, Shuyu Xiong, Jingjing Wang, Jun Chen, Zhuoting Zhu, Grace Brochert, Bo Zhang, Junjie Deng, Tianyu Cheng, Xiangui He, Xun Xu

**Affiliations:** 1 Department of Ophthalmology, Shanghai General Hospital, Shanghai Jiao Tong University School of Medicine, National Clinical Research Center for Eye Diseases, Center of Eye Shanghai Key Laboratory of Ocular Fundus Diseases, Shanghai Engineering Center for Visual Science and Photomedicine, Shanghai, China; 2 Department of Clinical Research, Shanghai Eye Disease Prevention and Treatment Center, Shanghai Eye Hospital, Shanghai Vision Health Center & Shanghai Children Myopia Institute, Shanghai, China; 3 Centre for Eye Research Australia, Ophthalmology, University of Melbourne, Melbourne, VIC Australia

**Keywords:** Refractive errors, Paediatrics, Epidemiology

## Abstract

**Objectives:**

To characterize choroidal vascular changes in children with different refractive status.

**Methods:**

A study including 5864 children aged 6–9 years was performed to investigate the choroidal vascular index (CVI) in myopic, emmetropic and hyperopic eyes. Each participant had a comprehensive ocular examination with cycloplegic autorefraction performed, axial length (AL) measured and Swept Source-Optical Coherence Tomography (SS-OCT) scans acquired. Choroidal thickness (ChT) was measured by built-in software, and CVI was calculated using a previously validated self-developed algorithm.

**Results:**

The mean ChT and CVI were 275.88 ± 53.34 μm and 34.91 ± 3.83 in the macula region, and 191.96 ± 46.28 μm and 32.35 ± 4.21 in the peripapillary region. CVI was significantly lowest for myopes, followed by emmetropes and hyperopes (*P* < 0.001). CVI varied between different sectors separated by the Early Treatment of Diabetic Retinopathy Study (ETDRS) grid (*P* < 0.001). Macular CVI decreased horizontally from nasal to temporal quadrant with lowest in center fovea, and vertically from superior to inferior quadrants. Peripapillary CVI was highest in the nasal and lowest in the inferior sector. Multiple regression showed that spherical equivalent (SE), AL, intraocular pressure (IOP), ChT, age, and gender were significantly related to CVI (*P* < 0.05).

**Conclusions:**

In children, the distribution of CVI in the posterior pole is not uniform. A decreased CVI was observed from hyperopia to myopia and was associated with decreased SE, elongated AL, and choroidal thinning. Further study of changes in CVI during myopia onset and progression is required to better understand the role of the choroidal vasculature in myopia development.

## Introduction

Myopia is the most common refractive error. It has a substantial burden on the public health system and has a significant impact on quality of life [1, 2]. Despite extensive research, the underlying pathogenesis of myopia is not yet fully understood. Previous studies have suggested a significant association between choroidal thickness (ChT) and myopia [3–7]. The choroid is a highly vascularized tissue located between the sclera and the retina. Changes in ChT can lead to changes in choroidal blood flow (ChBF) [8]. Studies have demonstrated that reduced ChBF may result in scleral hypoxia, leading to the extracellular matrix remodeling observed in myopia [9–11]. Some animal and population studies have demonstrated decreased choroidal blood perfusion in myopes [12–15]. It has been suggested that changes in choroidal vascular, maybe a reliable imaging biomarker in the development of myopia [16], as the choroidal vasculature is responsible for the delivery of oxygen and nutrients to the outer retina [8].

The measurement of ChBF remains challenging without a direct, efficient, and non-invasive method of visualization of the vascularity and blood flow in the choroid. Optical coherence tomography (OCT) provides a cross-sectional view of the ocular structures in vivo [17]. Swept Source-Optical Coherence Tomography (SS-OCT) has enabled increased visualization of the choroid [18]. SS-OCT has great potential for non-invasive quantitative assessment of the choroidal layer by providing higher penetration and resolution, to give a more detailed visualization on the choroidal vessels [19, 20]. Recently, an image binarization method was proposed to segment the choroidal scans to distinguish between the choroidal luminal and stromal area. Choroidal vascular index (CVI), defined as the ratio of luminal area to total choroidal area, can be used to evaluate the ChBF [21–23].

Studies on CVI are increasing in various ocular diseases [24–28], but there are very few in myopia, particularly in children with myopia. In this study, a previously validated deep learning method, RefineNet [29], was applied to a large-scale population study to segment the choroidal vessels automatically and accurately from SS-OCT images. The morphological and vascular characteristics of the choroid in children with different refractive status were evaluated by CVI. The aim is to better explore the distribution of CVI over different refractive error status, and the influential factors on CVI.

## Subjects and methods

### Study population

This is a prospective school-based study (Shanghai Time Outside to Reduce Myopia, STORM) conducted in Shanghai, China. The STORM study was conducted from 2016 to 2018 and the study methodology was previously reported [30]. A cluster sampling method was used to recruit 6295 students aged 6 to 9 years from 24 primary schools. Participants were excluded if they had systemic or ocular disease, such as strabismus, or amblyopia, and if participants used any myopia intervention.

The STORM study was approved by the Ethics Committee of Shanghai General Hospital (Reference number: 2016KY138) and adhered to the tenets of the Declaration of Helsinki. Written informed consent of participants was obtained from their parents/caregivers. Prior to all measurements, oral assent was obtained from each participant. The trial was registered with ClinicalTrials.gov, identifier: NCT02980445.

### Ocular examination and measurements

All the participants completed a standardized questionnaire, had a slit-lamp examination, and had several measurements including axial length (AL), IOP, cycloplegic autorefraction, and ChT and ChBF using SS-OCT.

The IOL Master 500 (version 5.02; Carl Zeiss, Jena, Germany) was used to measure AL before cycloplegia. This was repeated three times and if the difference between any two measurements was >0.02 mm, it was further repeated. Before cycloplegia, the IOP was measured using a non-contact tonometer (NT-1000, Nidek, Tokyo, Japan). The slit-lamp examination was performed to ensure that the participants’ iridocorneal angle was suitable for cycloplegia and to rule out the presence of any ocular pathology. For cycloplegia, a drop of 0.5% proparacaine hydrochloride (Alcaine; Alcon, Fort Worth, TX, USA) was instilled in each eye. After 15–20 s, two drops of 1% cyclopentolate (Cyclogyl; Alcon, Fort Worth, TX, USA) were instilled in each eye at an interval of 5 min. After forty minutes, both eyes were checked for dilation and pupillary response to light. If necessary, a third drop of cyclopentolate was instilled. Cycloplegia was considered complete if the pupil size was dilated to 6 mm or greater and there was no pupil light reflex. Refraction was measured by a desk-mounted autorefractor (KR-8900; Topcon, Tokyo, Japan) with at least three measurements recorded. If any two measurements varied by more than 0.50D, the readings were discarded and the eye was remeasured. Meanwhile, parents/caregivers completed an online questionnaire for demographic purposes.

### SS-OCT image acquisition

After cycloplegia, SS-OCT (model DRI OCT-1 Atlantis, Topcon, Tokyo, Japan) was performed with a 12-line radial scan pattern centered on the fovea and optic disc and a resolution of 1024 for each line. The radial scan was separated by 15° to capture OCT images and each scan line was 9 mm long. Each image included the overlapping of 16 consecutive B -scans. Participants’ SE, AL, and curvature radius (CR) were inputted into the software to correct for optical magnification factors. The measurements were taken between 10:00 and 15:00 to minimize the confounding effect of diurnal variation [31]. Scans were retaken if the quality was poor, defined as a signal strength index <60 or image quality <90 or black lines on the image.

### Measurement of ChT and CVI

The segmentation of OCT layers and the construction of topographic maps were done using the built-in software, which was checked and corrected manually by the OCT technician. Previously this has been demonstrated to be efficient and there was good repeatability of the technician [3]. The detailed analysis method for ChT has been described previously [4]. ChT was defined as the distance between Bruch’s membrane and the choroidal-scleral interface. The ETDRS grid was centered on the fovea and optic disc, separating these two regions into nine sectors by three concentric circles. The diameters for the inner circle (central foveal circle), middle circle (parafoveal circle), and outer circle (perifoveal circle) were 1 mm, 3 mm, and 6 mm, respectively, and were further subdivided into superior, inferior, temporal, and nasal quadrants. The average regional ChT of the ETDRS grid was calculated by the built-in software.

**Fig. 1:**
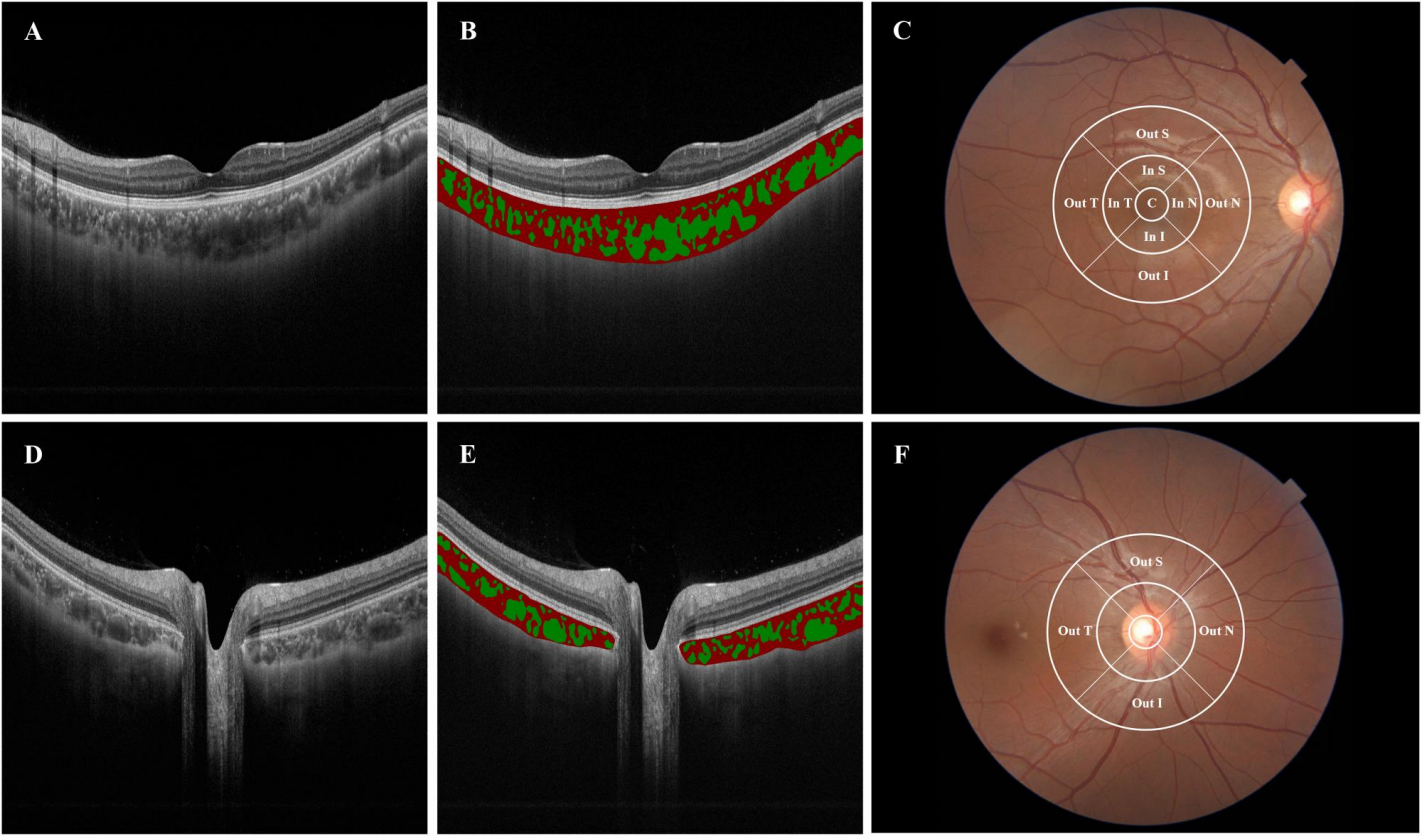
Choroidal Vascular Index (CVI) maps captured by SS-OCT in the macular and peripapillary region. Raw SS-OCT images were taken centered on the macula (**A**) and optic disc (**D**). The choroidal vessels were visualized on SS-OCT images and segmented by a deep learning method [29], in the macular region (**B**) and peripapillary region (**E**). Choroidal vessels were illustrated in green and choroidal matrix in red. The ETDRS grid was applied to show the CVI of each sector in the macular region (**C**) and peripapillary region (**F**). In N, In S, In T, In I corresponded to the inner circle of the ETDRS grid in the nasal, superior, temporal, and inferior sector. Out N, Out S, Out T, Out I referred to the outer circle of the ETDRS grid in the nasal, superior, temporal, and inferior sector. Since the inner and middle circles of the ETDRS grid in the peripapillary region were affected by the optic disc, only the CVI of the outer circle was included in the analysis.

CVI was assessed using an automated algorithm previously validated [29], which showed a high degree of vessel segmentation agreement with clinicians’ manual segmentation (Fig. 1B, E). The deep learning approach of RefineNet enabled higher segmentation accuracy in an end-to-end manner without pre-processing or post-processing techniques. The input SS-OCT image (Fig. 1A, D) was cropped into 500 × 500 patches due to the limited GPU memory. The framework was implemented in MATLAB R2017a and the average running time of RefineNet was 0.72 s per image. The CVI was calculated after the image was processed and outputted. The same ETDRS grid centered on both the fovea and optic disc was applied to the separation of CVI (Fig. 1C, F).

In the macular region, all nine sectors of ChT and CVI were included in the analysis. In the peripapillary area, only four sectors of the perifoveal circle were used, as the absence of choroidal tissue and the irregular topography map of the optic disc yielded unreliable ChT and CVI measurements.

### Statistical analysis

All statistical analyses used SPSS (version 25.0; IBM Co, Armonk, New York, USA). Only the data from the right eye were used in the final analysis. Based on cycloplegic autorefraction results, the SE was calculated by the sum of the sphere and half of the cylinder. Hyperopia, emmetropia and myopia were defined based on a SE ≥ +1.00 D, −0.50 D < SE < +1.00 D, and SE ≤ −0.50 D, respectively [32].

The data distribution was examined by the Kolmogorov–Smirnov test. Continuous variables conformed to or approximated to the normal distribution and were expressed as means ± standard deviation (SD). Meanwhile, categorical data were reported as proportions. Discrepancies among different refractive groups were evaluated by one-way ANOVA and the Bonferroni method was used for post hoc tests. The categorical variables were compared with chi-square tests. Repeated-measures analysis of variance (RM-ANOVA) was performed to compare the CVI in different sectors. The Greenhouse–Geisser correction was applied when the sphericity assumption was violated. Spearman’s correlation was used to calculate the correlation between CVI and ocular biometric variables. Linear regression was applied to determine the association between CVI and ocular biometric variables in different refractive groups. Multiple regression analysis was conducted to explore the independent factors for CVI. A two-sided *P* < 0.05 was considered statistically significant.

## Results

### General characteristics

Of the initial 6295 participants, 5864 (93.15%) completed all components of the ocular examination with high-quality SS-OCT images. 168 participants were excluded due to poor cooperation for SS-OCT imaging, and 263 were excluded due to poor image quality. Participants excluded and included in the analysis were comparable from baseline demographics such as age and sex (*P* > 0.05). The mean age of the participants was 7.24 ± 0.60 years (range 6–9 years) and 3081(52.5%) were boys. The mean SE was 1.01 ± 1.00 D and the mean AL was 22.87 ± 0.76 mm. The mean ChT in macular and peripapillary region was 275.88 ± 53.34 μm and 191.96 ± 46.28 μm. The general characteristics of the enrolled participants are detailed in Table 1.

**Table 1:** Demographics of participants in Different Refractive Groups^a^.

Parameters	Total (*n* = 5864)	Myopes (*n* = 396)	Emmetropes (*n* = 1909)	Hyperopes (*n* = 3559)	*P* value
Boys, *N (%)*	3081 (52.5)	233 (58.8)	1030 (54.0)	1818 (51.1)	0.004*
Age, years	7.24 ± 0.60	7.53 ± 0.52	7.35 ± 0.58	7.15 ± 0.59	<0.001^†^
Height, cm	125.09 ± 9.98	127.86 ± 6.50	125.99 ± 8.90	124.38 ± 10.66	<0.001^†^
Weight, kg	26.55 ± 6.48	28.21 ± 6.82	27.03 ± 6.16	26.15 ± 6.41	<0.001^†^
BMI, kg/m^2^	16.77 ± 2.71	17.09 ± 2.98	16.87 ± 2.73	16.69 ± 2.67	0.011^†^
SE, D	1.01 ± 1.00	−1.40 ± 0.91	0.49 ± 0.33	1.56 ± 0.65	<0.001^†^
AL, mm	22.87 ± 0.76	23.88 ± 0.72	23.10 ± 0.64	22.64 ± 0.68	<0.001^†^
IOP, mmHg	15.22 ± 3.45	15.59 ± 3.20	15.40 ± 3.56	15.09 ± 3.41	0.003^†^
ChT, μm					
Macular region	275.88 ± 53.34	243.00 ± 49.23	268.56 ± 51.63	283.42 ± 52.71	<0.001^†^
Peripapillary region	191.96 ± 46.28	169.19 ± 42.11	188.92 ± 44.86	196.09 ± 46.59	<0.001^†^

*IOP* intraocular pressure, *SE* spherical equivalent, *D* diopter, *AL* axial length, *ChT* choroidal thickness, *N* number.

^a^Data were presented as mean ± standard deviation unless otherwise indicated.

*Statistical significance was tested using Chi-Square tests.

^†^Statistical significance was tested using one-way ANOVA. The Bonferroni method was used for post hoc tests and the results showed that all pairwise comparisons except for BMI between the myopia and emmetropia groups, as well as IOP between the myopia and emmetropia groups and between the myopia and hyperopia groups, were statistically significant.

There were 396 (6.75%) myopes, 1909 (32.55%) emmetropes and 3559 (60.69%) hyperopes. Myopic participants were significantly older (*P* < 0.001) and had a higher BMI (*P* = 0.011) than emmetropic and hyperopic participants. Compared to emmetropes, myopes had a longer AL, higher IOP and thinner ChT, and hyperopes had a shorter AL, lower IOP and thicker ChT (Table 1).

### Analysis of Choroidal Vascularity Index

The mean CVI was 34.91 ± 3.83 in the macular region and 32.35 ± 4.21 in the peripapillary region. Both in the macular and peripapillary regions, the mean CVI was significantly lowest for myopes (32.60 ± 3.92 and 29.73 ± 4.01), followed by emmetropes (34.98 ± 3.71 and 32.42 ± 4.10) and hyperopes (36.05 ± 3.94 and 33.47 ± 4.48) (SFig. 1, all *P* < 0.001).

There were significant differences between boys and girls in the macular CVI (34.47 ± 3.85 for boys vs. 35.39 ± 3.75 for girls, *P* < 0.001) and peripapillary CVI (32.06 ± 4.22 for boys vs. 32.68 ± 4.18 for girls, *P* < 0.001) (sFig. 2). CVI decreased with age in both the macular and peripapillary regions (sFig. 2). However, there was no statistical difference in peripapillary CVI between 7-year-old and combined 8- and 9-year-old participants.

### Topographic variation of Choroidal Vascularity Index

After separating into nine sectors based on EDTRS grid, the CVI was significantly lower in all sectors in myopes and higher in hyperopes (sTable 1, all *P* < 0.001).

**Fig. 2:**
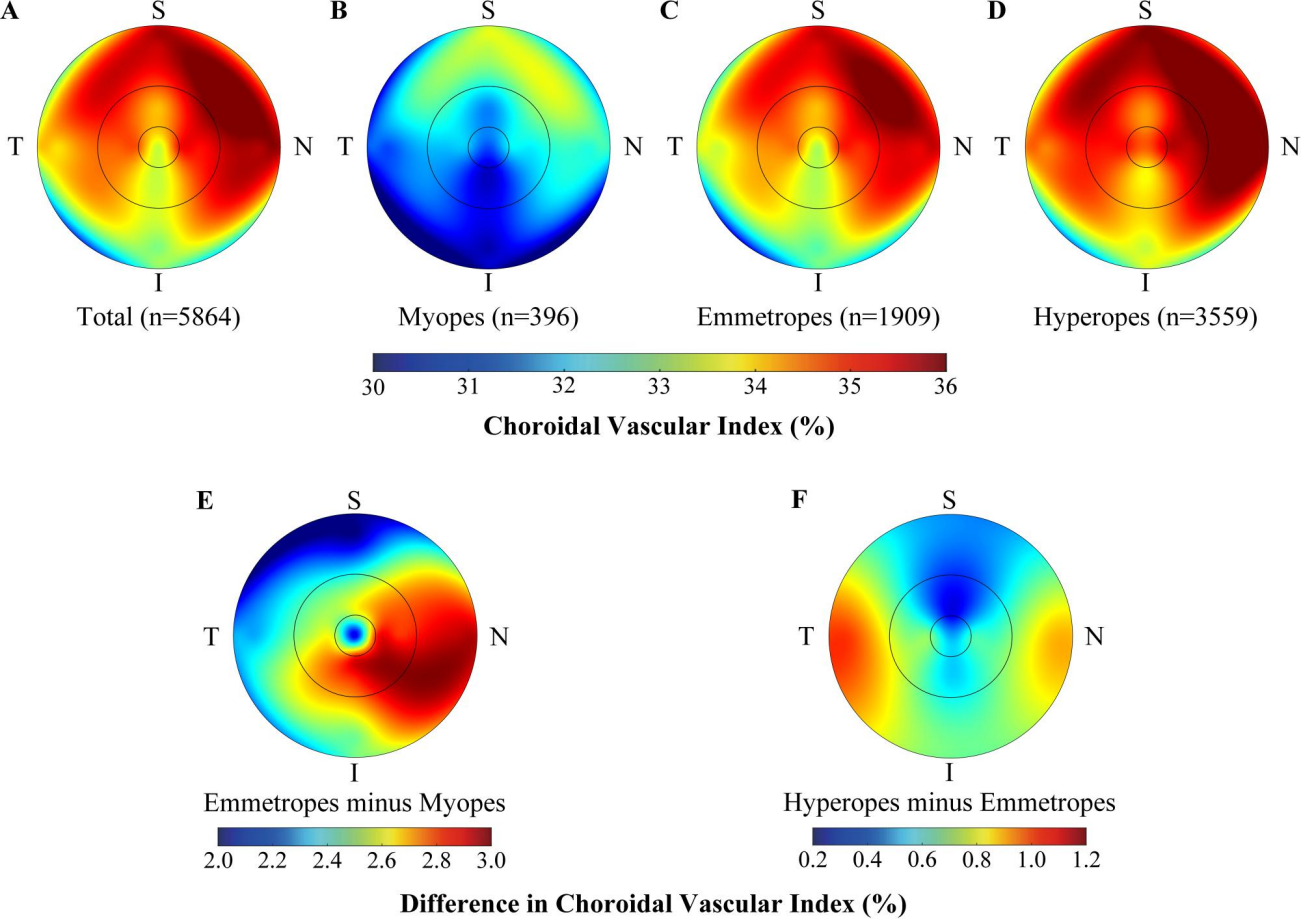
Topographic maps of Choroidal Vascular Index (CVI) in the macular region of participants with different refractive status. Distribution of CVI in macular region of overall participants (**A**), myopes (**B**), emmetropes (**C**), and hyperopes (**D**). Average difference in macular CVI between emmetropes and myopes (**E**), and between hyperopes and emmetropes (**F**). N nasal, S superior, T temporal, I inferior.

Variations of macular CVI in different sectors were presented in Fig. 2. For all participants, the CVI in the macular region differed significantly between the nine sectors (*P* < 0.001). The CVI in the macular region decreased horizontally from the nasal to the temporal quadrant, while vertically it decreased from the superior to the inferior quadrant (Fig. 2A). The center fovea, however, had the lowest CVI (33.39 ± 6.07) in the horizontal direction. The highest CVI was observed in the parafoveal nasal sector (35.69 ± 4.83) and the lowest in the parafoveal inferior sector (32.97 ± 4.41). The topographic maps of CVI for myopes, emmetropes and hyperopes were similar to the total participants (Fig. 2B, C, D). It was just that myopes had the lowest CVI overall in all nine sectors, while hyperopes had the highest CVI. The difference in macular CVI between emmetropes and myopes was greatest in the nasal quadrant and least in the superior quadrant (Fig. 2E). Meanwhile, the difference in macular CVI between hyperopia and emmetropes was smaller in all nine sectors, with the least difference in the perifoveal superior sector (Fig. 2F).

In the peripapillary region, the CVI in the nasal sector of the outer circle (31.71 ± 5.24) was highest, followed by the temporal (30.51 ± 5.12) and superior (29.26 ± 5.63) sector, while the inferior sector was the lowest (29.03 ± 5.72). Similar topographic variations were observed in participants with different refractive status (STable 1).

### Factors associated with Choroidal Vascularity Index

CVI in each sector of the EDTRS grid was positively correlated with ChT and SE, and negatively correlated with AL (sTable 2). The correlation coefficients ranged from 0.214 to 0.332 (all *P* < 0.01) between macular CVI in nine sectors and ChT in the corresponding region, from 0.096 to 0.175 (all *P* < 0.01) between CVI and SE, and from −0.193 to −0.144 between CVI and AL (all *P* < 0.01). Similarly, the correlation coefficients ranged from 0.344 to 0.463 (all *P* < 0.01) between peripapillary CVI in four sectors and ChT in the corresponding region, from 0.081 to 0.155 (all *P* < 0.01) between CVI and SE, and from −0.170 to −0.084 between CVI and AL (all *P* < 0.01).

**Fig. 3:**
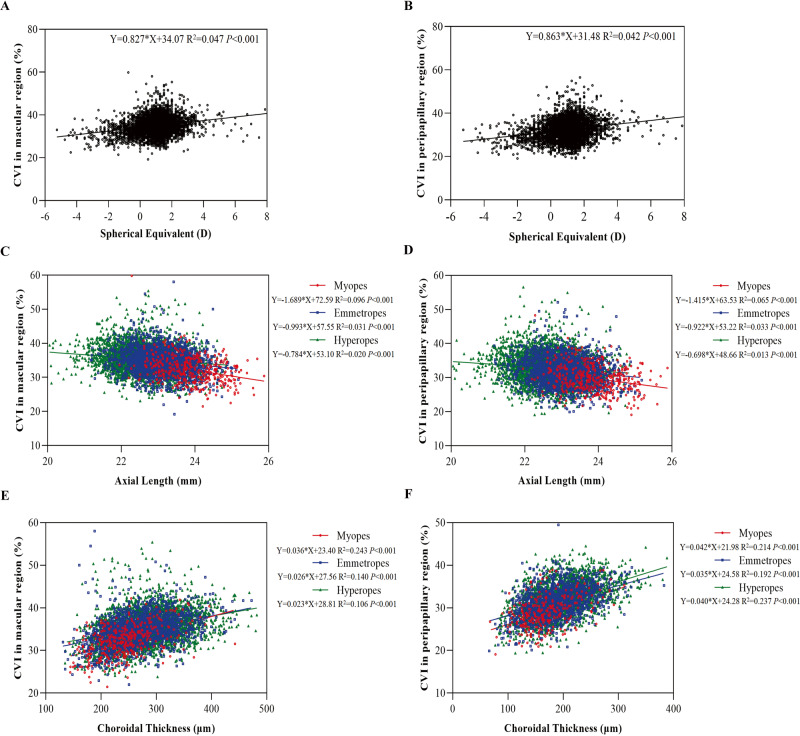
Linear regression between Choroidal Vascular Index (CVI) and different ocular biometric values. In the macular region, CVI against spherical equivalent (SE) (**A**), axial length (AL) (**C**), choroidal thickness (ChT) (**E**). In the peripapillary region, CVI against SE (**B**), AL (**D**), ChT (**F**).

A linear regression analysis between CVI and SE, and between CVI and AL/ ChT for the different refractive groups was seen in Fig. 3. There was a positive correlation between SE and CVI in macular (*R*^2^ = 0.047, *P* < 0.001, Fig. 3A), and peripapillary regions (*R*^2^ = 0.042, *P* < 0.001, Fig. 3B). AL was negatively associated with CVI in macular region (for myopes: *R*^2^ = 0.096; emmetropes: *R*^2^ = 0.031; hyperopes: *R*^2^ = 0.020, all *P* < 0.001, Fig. 3C). AL was also negatively associated with CVI in peripapillary region (for myopes: *R*^2^ = 0.065; emmetropes: *R*^2^ = 0.033; hyperopes: *R*^2^ = 0.013, all *P* < 0.001, Fig. 3D). The close association was found between macular CVI and ChT in myopes, emmetropes and hyperopes (*R*^2^ = 0.243, 0.140, 0.106, respectively, all *P* < 0.001, Fig. 3E), and between peripapillary CVI and ChT in myopes, emmetropes and hyperopes (*R*^2^ = 0.214, 0.192, 0.237, respectively, all *P* < 0.001, Fig. 3F).

Stepwise multiple linear regression models were constructed to investigate the association between the systemic and ocular factors with CVI. It showed that higher SE, shorter AL, higher IOP, thicker ChT, younger age, and girls were significantly (all *P* < 0.05) related to higher CVI in macular region (*R*^2^ = 0.175, Table 2). Similar results were noted for CVI in peripapillary region, in which higher SE, lower IOP, thicker ChT, younger age, and girls were significant (all *P* < 0.05) related to higher CVI in peripapillary region, with an *R*^2^ of 0.250. However, there was no significant relationship between AL and CVI in peripapillary region (*P* = 0.475).

**Table 2:** Multiple regression analysis of associated factors with Choroidal Vasculature Index (CVI) in macular and peripapillary region.

Independent variables	Unstandardized coefficients (95% CI)	Standardized coefficients	VIF	Variable’s *P* value	Equation’s *P* value^a^	*R*^2^
CVI in macular region						
Intercept	36.260 (31.702–40.819)	/	/	<0.001	<0.001	0.175
SE, D	0.340 (0.213–0.467)	0.090	1.569	<0.001		
AL, mm	−0.198 (−0.382 to −0.014)	−0.040	1.889	0.035		
IOP, mmHg	0.047 (0.017–0.077)	0.043	1.014	0.002		
ChT, μm	0.023 (0.021–0.025)	0.321	1.098	<0.001		
Age, years	−0.714 (−0.890 to −0.539)	−0.113	1.219	<0.001		
Gender (girls)	0.662 (0.437–0.886)	0.087	1.221	<0.001		
CVI in peripapillary region						
Intercept	27.804 (22.928–35.174)				<0.001	0.250
SE, D	0.314 (0.177–0.451)	0.082	1.529	<0.001		
AL, mm	−0.072 (−0.271 to 0.126)	−0.014	1.848	0.475		
IOP, mmHg	−0.047 (−0.079 to −0.015)	−0.043	1.029	0.004		
ChT, μm	0.038 (0.036–0.041)	0.466	1.086	<0.001		
Age, years	−0.278 (−0.471 to −0.085)	−0.043	1.067	0.005		
Gender (girls)	0.337 (0.093–0.581)	0.044	1.211	0.007		

^a^Stepwise multiple regression analysis was used.

## Discussion

This study represents a large-scale (*n* = 5864) pediatric population, with different refractive status, investigating the topographical characteristics and factors affecting CVI in macular and peripapillary regions. Our results demonstrated that myopic children had a lower CVI in all sectors of the ETDRS grid compared to emmetropic and hyperopic children, with the highest in the nasal perifoveal sector and the lowest in the inferior peripapillary sector. There was a decline in CVI for girls and with increasing age. CVI in macular and peripapillary regions was closely associated with ocular parameters such as ChT, AL, and SE. CVI decreased with ChT thinning, AL became elongated and myopia was more severe.

CVI was recently used for quantitative assessment of the choroidal vasculature and has been demonstrated to be a reliable biomarker [33–35]. It can be calculated manually or using an automated algorithm based on OCT images. Previous CVI-related studies were mostly conducted in adults and were focused only on the macular choroid [5, 15, 36, 37]. Our study found that the distribution of average CVI was similar to ChT in children with different refractive status [6], with CVI in both macular and peripapillary regions being highest in hyperopes, followed by emmetropes, and lowest in myopes. This was consistent with the findings of a previous study in anisomyopic young adults, where the CVI were smaller in the more myopic eyes in vertical and horizontal scans [15].

Previous studies reported inconsistent findings with topographic changes of CVI. A study in young adults showed that the highest CVI was found in the nasal quadrant, followed by the inferior, temporal, and superior quadrant [20]. Meanwhile, another study in healthy young adults showed that CVI was lowest in the fovea and increased towards the periphery, with the highest CVI in the nasal quadrant and the lowest CVI in the temporal quadrant [37]. Agrawal et al. similarly found that the CVI in the subfoveal scans was lower than in central macular and total macular scans [38]. Our study, consistent with previous reports, shows the CVI in macular region decreased from the nasal to the temporal quadrant while the CVI in center fovea was the lowest. The lower center fovea CVI may be due to the greater proportion of large choroidal vessels in the periphery compared to the center fovea; the higher CVI in the nasal compared to the temporal quadrant may be due to the greater abundance of non-vascular smooth muscle cells in the temporal rather than the nasal choroid [20, 37]. However, vertically, our study found a decrease in CVI in the macula from the superior to the inferior quadrant, which was comparable to the findings of Wu et al. [15] but differed from the results of Yazdani et al. [37]. This may be explained by different study populations or scanning patterns and still needs further investigation.

When further exploring topographic differences in macular CVI between children with different refractive status, we found greater differences in the nasal quadrant of the macula between myopic and emmetropic children, and greater differences in the temporal quadrant between hyperopic and emmetropic children. Previous studies indicated that the thinning of choroidal in myopes was more pronounced in the central fovea [39] which contrasts with the observed CVI change observed in our study. Several factors may explain why the decrease in CVI is not most significant in the central fovea. Firstly, the reduction in choroidal luminal area and stromal area was not uniform. Near the central fovea, microstructural variations could result in a relatively smaller decrease in luminal area compared to the stromal area [5, 37]. Additionally, it is important to note that some of these studies used different cycloplegic agents, which may affect the measurement of CVI. Therefore, our hypothesis that myopes have a greater decrease in ChT but less decrease in CVI in the central fovea may need to be reevaluated in future studies that account for the potential impact of different cycloplegic agents on CVI measurements. Further research is also needed to investigate the mechanisms underlying the observed topographic differences in macular CVI between children with different refractive status.

Previous studies on CVI in the peripapillary region were conducted mostly in glaucoma patients [25, 40, 41] and no studies examined the distribution of CVI in the peripapillary region in children. We found the peripapillary CVI in nasal sector was highest, followed by the temporal and superior sector, while the inferior sector was the lowest, which was similar to the CVI distribution of the control population in the study by Simsek et al. [28]. The peripapillary region has unique anatomical and physiological significance [42] with analysis of CVI in the peripapillary region being useful in assessing the blood supply to the optic nerve.

Our study indicated that SE, AL, IOP, ChT, and age were independent factors of CVI. Some studies reported no significant relationship between CVI and ocular parameters such as SE and AL [22, 36], while others showed a negative correlation between luminal and stromal areas and AL [5, 43]. Together the high correlation between CVI and ChT and since ChT correlated positively with SE although negatively with AL in previous studies [4, 6, 39], it is suggested the smaller SE and longer AL in our study had lower CVI. Very few studies have explored the relationship between age and CVI. Agrawal et al. reported no significant correlation between CVI and age [22]. However, a large number of studies on ChT indicated that ChT decreased significantly with age [39, 44, 45]. Our results found a decreasing trend in CVI with age in the macular and peripapillary regions. These results need to be considered with caution since a narrow age range was included in this study.

A positive correlation between CVI and ChT has been demonstrated [22], but it is unclear if a causal relationship. An animal study used vasodilators to increase ChBF and found that myopia progression was slowed, axial length elongation was suppressed, and scleral hypoxia was improved in guinea pigs [46]. Therefore, it is suggested that choroidal thinning in myopes is most likely a result of the reduction in ChBF leading to a hypoxic environment, which in turn leads to AL elongation, thus promoting myopia progression.

This study has several limitations. Firstly, it is a cross-sectional study using baseline data from the STORM study, providing only evidence for a correlation between reduced ChBF and myopia. Future longitudinal studies are needed to investigate the relationship between ChBF and myopia onset and progression. Secondly, different algorithms are used for choroidal segmentation, making comparisons between studies difficult [47–50]. However, our previously validated algorithm was experimentally demonstrated to be highly similar to the physician’s manual segmentation, providing accurate information on the choroidal vasculature. Thirdly, we acknowledge the potential impact of cyclopentolate on both Choroidal Vascularity index (CVI) measurements and axial length (AL) measurements, as studies have reported thinning of the choroid following the instillation of cyclopentolate [51] and changes in ChT caused by the use of cyclopentolate will result in corresponding changes in AL measurements [52]. While we did not specifically address these issues in our study, they are important considerations for future studies in this area. Finally, as the study was conducted in Chinese children aged 6–9 years, the results are population-, region-, and age-specific. Therefore, caution should be exercised when generalizing the results to other populations.

## Conclusion

In conclusion, we characterized the distribution of CVI in the posterior pole in children with different refractive status. Children with myopia have reduced ChT, and a decreased CVI compared to emmetropic and hyperopic eyes. The decrease in CVI from emmetropia to myopia was greatest in the nasal quadrants of the macula region. Reduced CVI was positively associated with higher refractive errors, AL elongation, and choroidal thinning. We therefore hypothesize that changes in ChBF may be involved in the thinning of ChT during myopia onset and shift. CVI is expected to be an adjunctive assessment tool for children with myopia, applied in large-scale longitudinal studies of school-aged children to clarify the causal relationship between ChT and ChBF, which may help improve our understanding of the etiology of myopia.

## Summary

### What was known before

- Choroidal thickness thins in people with myopia. Choroidal blood flow is closely related to choroidal thickness.
- The binarization technique enabled the evaluation of the Choroidal vascularity index to reflect choroidal blood flow.

### What this study adds

- Choroidal blood flow is unevenly distributed in the posterior pole.
- Choroidal vascularity index gradually decreases from hyperopia to emmetropia and then to myopia.
- Reduced CVI was positively associated with higher refractive errors, AL elongation, and choroidal thinning.

### Supplementary information

supplementary

## Data Availability

The data used and/or analyzed during the current study are available from the corresponding author on reasonable request.
