# Supplementary material for: Macular and peripapillary Choroidal Vascularity Index in children with different refractive status
Source: Eye (Lond). 2023 Sep 28;38(3):606–13. doi: 10.1038/s41433-023-02743-1 (PMC10858217; doi:10.1038/s41433-023-02743-1)
Supplement: Supplementary file 1 — supplementary [file 41433_2023_2743_MOESM1_ESM.pdf]

Supplementary Table 1. Comparison of Choroidal Vascular Index in myopes, emmetropes, and hyperopes participants

Parameters	Total (n=5864)	Refractive status			*P* value*
		Myopes (n=396)	Emmetropes (n=1909)	Hyperopes (n=3559)	
**CVI in macular region, %**					
**Center foveal**	33.39±6.07	31.15±6.01	33.24±5.85	34.73±6.14	<0.001
**In N**	35.17±5.49	32.15±5.64	34.98±5.19	35.61±5.52	<0.001
**In S**	34.12±5.06	31.53±4.93	34.11±4.85	34.42±5.11	<0.001
**In T**	34.69±5.44	31.87±5.66	34.44±5.20	35.14±5.44	<0.001
**In I**	33.41±5.27	30.43±5.28	33.28±5.06	33.81±5.28	<0.001
**Out N**	35.69±4.83	32.42±4.69	35.35±4.72	36.24±4.75	<0.001
**Out S**	35.54±4.32	33.39±4.32	35.39±4.21	35.85±4.30	<0.001
**Out T**	33.89±4.80	31.13±5.10	33.42±4.56	34.45±4.76	<0.001
**Out I**	32.97±4.41	30.25±4.49	32.72±4.28	33.40±4.35	<0.001
**CVI in peripapillary region, %**					
**Out N**	31.71±5.24	28.72±5.05	31.25±5.05	32.29±5.22	<0.001
**Out S**	29.26±5.63	26.39±5.07	28.71±5.33	29.87±5.72	<0.001
**Out T**	30.51±5.12	28.50±5.30	30.17±5.00	30.91±5.11	<0.001
**Out I**	29.03±5.72	26.92±5.35	28.76±5.40	29.42±5.86	<0.001

Choroidal Vascular Index (CVI) was presented as mean ± SD. In, Inner; Out, Outer.

* Statistical significance was tested using one-way ANOVA. Bonferroni method was used for post hoc tests.

Supplementary Table 2. Spearman Correlation Coefficient (ρ) between ocular biometrics and Choroidal Vascular Index (CVI) in different regions

	ChT [a]	SE	AL
**CVI in macular region**			
**Center foveal**	0.315*	0.096*	-0.165*
**In N**	0.307*	0.126*	-0.183*
**In S**	0.271*	0.105*	-0.144*
**In T**	0.331*	0.137*	-0.171*
**In I**	0.332*	0.126*	-0.182*
**Out N**	0.214*	0.163*	-0.191*
**Out S**	0.254*	0.114*	-0.147*
**Out T**	0.301*	0.175*	-0.176*
**Out I**	0.244*	0.150*	-0.193*
**Total**	0.394*	0.184*	-0.219*
**CVI in peripapillary region**			
**Out N**	0.344*	0.152*	-0.170*
**Out S**	0.404*	0.155*	-0.157*
**Out T**	0.384*	0.110*	-0.137*
**Out I**	0.463*	0.081*	-0.084*
**Total**	0.485*	0.179*	-0.193*

[a] ChT in correlation analysis is the part matching the region of CVI, such as ChT in center foveal and CVI in center foveal.

* $P < 0.01$ (2-tailed) by Spearman's correlation analysis.

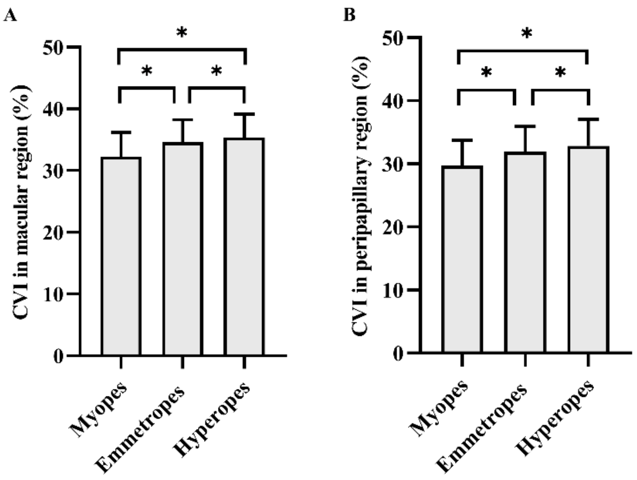

Supplementary Fig 1. Distribution of Choroidal Vasculature Index (CVI) in the macular region (A) and peripapillary region (B) of all participants with different refractive status.

The data are presented as bar graphs with mean values and standard deviations (SDs).

* Statistical analysis was by one-way ANOVA with Bonferroni method used for post hoc tests.

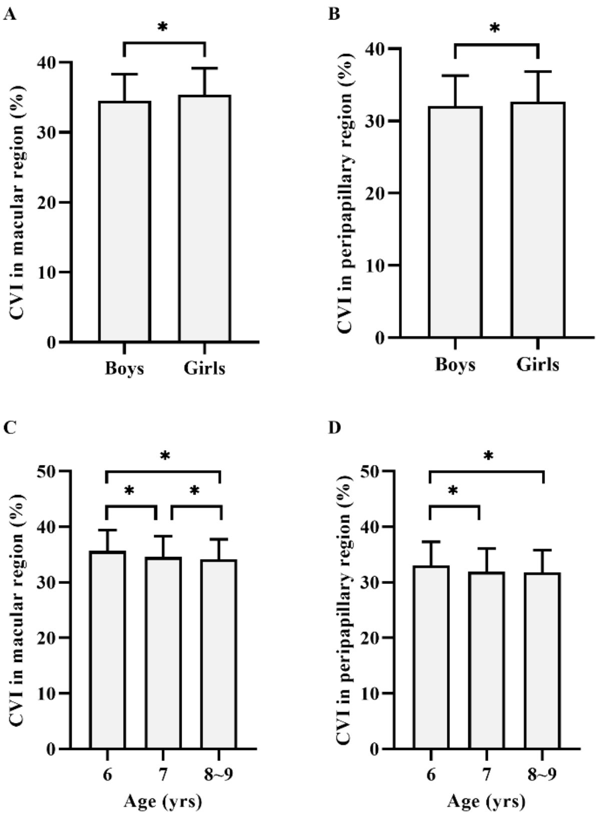

Supplementary Fig 2. Differences of Choroidal Vascular Index (CVI) in macular and peripapillary region in different genders (A and B) and age groups (C and D).

As only 4 individuals were 9 years old, 8- and 9-year-old children were combined into a single age group to increase the statistical power.

The data are presented as bar graphs with mean values and standard deviations (SDs).

*Statistical significance of gender differences with a t test. Statistical significance of age differences was tested using one-way ANOVA. Bonferroni method was used for post hoc tests.
